# Frequency and Causes of Deferral among Blood Donors Presenting to Combined Military Hospital Multan

**DOI:** 10.7759/cureus.6657

**Published:** 2020-01-14

**Authors:** Hamid Iqbal, Asma Tameez Ud Din, Asim Tameez Ud Din, Farooq Mohyud Din Chaudhary, Muhammad Younas, Abdur Jamil

**Affiliations:** 1 Hematology, Combined Military Hospital Multan, Multan, PAK; 2 Internal Medicine, Rawalpindi Medical University, Rawalpindi, PAK; 3 Gastroenterology, Nishtar Medical University & Hospital, Multan, PAK; 4 Chemical Pathology, Combined Military Hospital Multan, Multan, PAK; 5 Internal Medicine, Central Michigan University, Saginaw, USA

**Keywords:** blood donors, deferral, anemia

## Abstract

Background & Aim

It is of great importance to carefully choose appropriate donors according to strict eligibility criteria, so as to guarantee an adequate and safe blood supply. The aim of this study was to determine the rate of deferral in blood donors and evaluate the different causes of deferral in Multan.

Materials & Methods

This prospective study was carried out at the Blood Bank of Combined Military Hospital (CMH) Multan. All donors who came for the donation of blood from 1st February to 30th September 2019 were evaluated after taking their consent. The data was analyzed to determine the frequency and causes of deferral using Statistical Package for the Social Sciences (SPSS) version 20.

Results

Among 3348 individuals presenting for blood donation, 433 (12.9%) were deferred (427 males and only six females). The mean age of deferred individuals was 28.96 + 6.42 years. The youngest individual was 18 years, while the eldest one was 51 years of age. Almost 65% of the individuals were less than 30 years of age. The most frequent cause of deferral was low hemoglobin. Anemia was the leading cause of deferral in more than half of the individuals (*n *= 221). Hepatitis C virus (HCV) infection was the second most frequent cause of deferral, seen in 83 (19.2%), followed by hepatitis B virus (HBV) infection (*n *= 49, 11.3%), syphilis (*n *= 36, 8.3%), thrombocytopenia (*n *= 18, 4.2%), and active infection (*n *= 14, 3.2%). Other rarer causes included early donation, thrombocytosis, polycythemia, pancytopenia, malaria, allergies, insulin, and tuberculosis.

Conclusion

Deferral for blood donation is a significant problem in Multan and accounts for almost 13% of all prospective blood donors. Our results stress the importance of addressing the problem of anemia which is the most prevalent cause of temporary deferral for blood donation in this region of the world.

## Introduction

Blood transfusion is an essential life-saving procedure. Its inpatient use for a variety of medical and surgical etiologies is increasing day by day. Approximately 85 million units of red blood cells (RBCs) are transfused globally per year [1]. According to another report, there is an overall 85.8% increase in the blood transfusion for the patients admitted in the hospitals from 2000 to 2013 [2]. This has led to an increased demand for blood donors to meet this requirement. Apart from this, there are significant benefits to blood donors’ health. There is a reported decrease in cardiovascular risk in blood donors as compared to non-donors [3-4]. Also, it is found to be protective against cancer, insulin resistance, and plaque rupture [5-8]. 

Blood donors could be voluntary, paid, or replacement donors who are usually close contacts of patients [9]. For the welfare and safety of donors and recipients, a thorough clinical and laboratory screening is done. On the basis of these results, some of the donors can be temporary or permanently deferred. This is not only frustrating for the donors but also for the healthcare professionals and societies involved in blood collection. The common causes of temporary deferral include low hemoglobin, infections including malaria, and duration of last blood donation less than three months. The donors could be permanently deferred due to underlying chronic conditions [10]. 

Multiple studies are conducted in different regions of the world to investigate the rates of blood deferral and its common etiologies. Our study aimed to comprehensively analyze the deferral rates, the different causes of deferral and donor characteristics of individuals presenting to Combined Military Hospital (CMH), Multan which is one of the largest centers in South Punjab, Pakistan.

## Materials and methods

This prospective study involved all individuals who presented for blood donation from February 1, 2019 to September 30, 2019 at the Blood Bank of Combined Military Hospital (CMH), Multan after taking approval from the Institutional Ethical Review Board. Informed consent was taken from all blood donors. 

All persons coming for blood donation were first counseled upon the process of blood donation by trained medical professionals. Then using a standardized questionnaire, the socio-demographic characteristics (age, gender, marital status, address) of participants were recorded. Inquiry about past diseases was made from all individuals. Measurements for weight and blood pressure were made. A complete blood examination was then undertaken. 

Testing for Human Immunodeficiency Virus (HIV), hepatitis B virus (HBV), hepatitis C virus (HCV), malaria and syphilis was done. Participants who do not fulfill the selection criteria for the donation were then deferred either temporarily or permanently depending on the reason for the deferral. The data was analyzed to determine the frequency and causes of deferral using SPSS version 20.

## Results

Out of the 3348 individuals who came for blood donation during the study period, 433 (12.9%) were deferred. The majority of deferred individuals were males (*n*=427, 98%). There were only 6 female participants who were deferred. The mean age of the deferral group was 28.96 years with a standard deviation (SD) of 6.42 years. The youngest individual was 18 years, while the eldest one was 51 years of age. The majority of the deferred individuals belonged to the 21-30 years age group. Almost 65% of the individuals were less than 30 years of age. The different characteristics of the deferred participants are shown in Table 1.

**Table 1:** Characteristics of deferred individuals (N = 433)

Characteristics	N (%)
Age (years)	
Mean	28.96
Standard Deviation	6.42
Range	18-51
Gender	
Male	427 (98.6)
Female	6 (1.4)
Age Groups (years)	
< 20	27 (6.2)
21-30	194 (44.8)
31-40	101 (23.3)
41-50	16 (3.7)
> 50	1 (0.2)
Hemoglobin (g/dl)	
Mean	13
Standard Deviation	1.72
Range	7.2-17.5

The most frequent cause of deferral was low hemoglobin. Anemia was the leading cause of deferral in more than half of the individuals (*n *= 221, 50.3%). Hepatitis C virus (HCV) infection was the second most frequent cause of deferral, seen in 83 (19.2%), followed by hepatitis B virus (HBV) infection (*n *= 49, 11.3%), syphilis (*n *= 36, 8.3%), thrombocytopenia (*n *= 18, 4.2%), and active infection (*n *= 14, 3.2%). Other rarer causes included too early donation, thrombocytosis, polycythemia, pancytopenia, malaria, allergies, insulin, and tuberculosis. The different causes of deferral are listed in Table 2.

**Table 2:** Causes of deferral among blood donors (N = 433) *Anemia defined as Hemoglobin <13 g/dL in males, while <12 g/dL in females ** Thrombocytopenia defines as Platelet count < 150,000 /microliter *** Polycythemia defined as Hemoglobin >18 g/dL in males, while >16 g/dL in females **** Too early donation defined as last donation interval of less than 3 months HCV, hepatitis C virus; HBV, hepatitis B virus; TB, tuberculosis

Causes	n (%)
Anemia*	218 (50.3)
HCV	83 (19.2)
HBV	49 (11.3)
Syphilis	36 (8.3)
Thrombocytopenia**	15 (3.5)
Active Infection	14 (3.2)
Anemia + Thrombocytopenia	3 (0.7)
HBV + Syphilis	3 (0.7)
Malaria	3 (0.7)
Polycythemia***	2 (0.5)
HCV + Syphilis	1 (0.2)
Too early Donation****	1 (0.2)
Thrombocytosis	1 (0.2)
Pancytopenia	1 (0.2)
Allergy	1 (0.2)
Insulin dependent diabetes mellitus	1 (0.2)
TB	1 (0.2)

## Discussion

Blood donor selection is a key step in the transfusion process and usually comprises multiple checkpoints to ensure the safety of donors as well as the recipients. This process usually constitutes four steps. The first step includes sharing of knowledge about common infections that are transmissible during transfusion and other risks for a donor. This is followed by a donor health questionnaire that is completed by the donor and an interview by a permitted health professional (HP). Finally, the health of the donor is evaluated on the basis of physical and laboratory test results, which leads to acceptance or deferral of the donors. The deferral can be temporary or permanent depending upon the underlying condition. In our study, we aimed to find the frequency and common causes of deferrals among the blood donors [11].

Our study demonstrated a deferral rate of 12.9%. This is quite similar to a study conducted by Valerian et al. in northern Tanzania (12.7%) [12]. Another research study evaluating American Red Cross donation data showed a similar deferral rate (12.8%) [13]. But this is relatively high compared to some studies, conducted in the south region of Pakistan, Western India, and Malaysia which depicted deferral rates of 8.39%, 11.6%, and 5.6% respectively [14-16]. This contrast can be attributed to differences in sample size, demographic profile, and research methodology.

Low hemoglobin was found to be the most common cause of deferral (50.3%). This is comparable to other studies. A study carried out, using a structured questionnaire in 19 licensed blood banks of Pakistan, also manifested anemia as the leading cause of deferral (41%) [17]. Valerian *et al*. also demonstrated anemia as the common cause of temporary deferrals but the incidence was relatively lower (21.1%) [12]. Rabeya *et al.* showed 40.7% cases of deferrals due to low hemoglobin being predominantly females, but in our study, only 1.4% of the deferred cases were females [16]. In contrast to our results, a single-center study conducted in one of the blood centers in Pakistan showed therapeutic injections as the significant reason for blood donor deferrals, with low hemoglobin being second in the list [18]. An interesting study was conducted in South Pakistan to assess the deferral pattern on the basis of the peripheral count. It still showed anemia as the leading cause but depicted higher rates of polycythemia (3.3%) as opposed to our results (0.5%). By contrast, thrombocytopenia rates were higher in our study (3.5% versus 1.0%) [14].

The recommended cut-off for blood donation deferral is 12.5 g/dl, which implies that the donors with hemoglobin levels less than 12.5 g/dl are deferred irrespective of their demographic profile [19]. An extensive study was conducted in the United States to determine the association between the demographics and low hemoglobin in blood donor deferrals. It demonstrated female gender, advanced age in males and African-Americans, being significantly associated factors [20]. Our study comprised 98.6% males in the blood donor deferral group, and the mean age of the sample population was 28.96 years.

Hepatitis C infection was second among the common causes of a donor deferral (19.2%) but first in the list of infections. This was followed by hepatitis B virus infection (11.3%). Hepatitis B and C are highly prevalent diseases in Pakistan. Multiple studies showed an increased prevalence of these infections in blood donors [21-24]. Other causes indicated by our study include syphilis, active infection, and some rare disorders like thrombocytosis, polycythemia, malaria, and tuberculosis. In contrast to our study, Valerian et al showed syphilis as the second most common cause of temporary deferral, following anemia. The rates were relatively higher as compared to our study (9.3% versus 8.3%) [12].

This study has some limitations. As this was conducted in a single center in Pakistan, so the findings cannot be generalized. But as shown by Gillet *et al*., using different questionnaires and a careful review of blood donor deferrals, we can revamp the process of donor selection.

## Conclusions

In conclusion, blood screening deferral is a significant problem in our region. The most common cause of deferral found in our study was low hemoglobin, followed by infections like hepatitis C, hepatitis B, and syphilis. Other less common causes include early donation, thrombocytosis, polycythemia, pancytopenia, malaria, allergies, insulin, and tuberculosis. Thorough screening and evaluation of blood donors are essential for a safe blood transfusion.

## Figures and Tables

**Figure 1:**
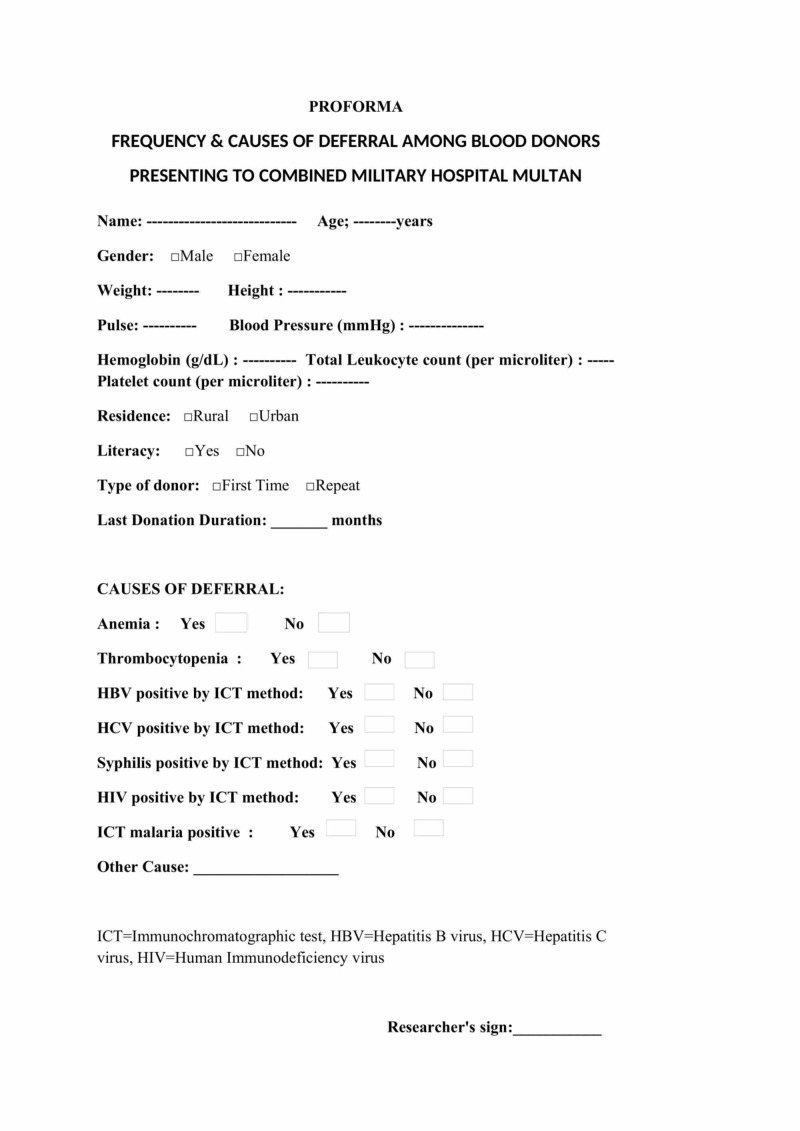
Questionnaire of the study ICT, immunochromatographic test; HBV, hepatitis B virus; HCV, hepatitis C virus; HIV, human immunodeficiency virus

## Appendices

A sample of the questionnaire used in our study is shown in Figure 1. 
